# Statistical Approaches for the Construction and Interpretation of Human Protein-Protein Interaction Network

**DOI:** 10.1155/2016/5313050

**Published:** 2016-08-25

**Authors:** Yang Hu, Ying Zhang, Jun Ren, Yadong Wang, Zhenzhen Wang, Jun Zhang

**Affiliations:** ^1^School of Life Science and Technology, Harbin Institute of Technology, Harbin 150001, China; ^2^Department of Pharmacy, Heilongjiang Province Land Reclamation Headquarters General Hospital, Harbin 150088, China; ^3^School of Computer Science and Technology, Harbin Institute of Technology, Harbin 150001, China; ^4^College of Bioinformatics Science and Technology, Harbin Medical University, Harbin 150081, China

## Abstract

The overall goal is to establish a reliable human protein-protein interaction network and develop computational tools to characterize a protein-protein interaction (PPI) network and the role of individual proteins in the context of the network topology and their expression status. A novel and unique feature of our approach is that we assigned confidence measure to each derived interacting pair and account for the confidence in our network analysis. We integrated experimental data to infer human PPI network. Our model treated the true interacting status (yes versus no) for any given pair of human proteins as a latent variable whose value was not observed. The experimental data were the manifestation of interacting status, which provided evidence as to the likelihood of the interaction. The confidence of interactions would depend on the strength and consistency of the evidence.

## 1. Introduction

Individual proteins cannot perform their biological functions by themselves, and actually they need to perform their functions in the biological process through interacting with other proteins [1]. Usually the interaction between two proteins means either they perform a biological function corporately or there is physical direct contact between them [2]. Most of the important molecular processes in cell, such as DNA replication, need to be performed by a large number of protein complexes. And these complexes are made up by the interactions between proteins. The study of PPIs is also considered to be a central problem in proteomics for living cells. Due to the dynamic interaction between proteins, the impact of surrounding environment should also be taken into account. The study of human PPI network can help to enhance the understanding of the disease but also provide a theoretical foundation for finding new treatment.

With the continuous progress and development of high-throughput experimental technology, more and more large quantities of interactions between human proteins had been confirmed by a variety of experimental methods. And many kinds of biological interaction networks have been investigated [3–7]. However, current high-throughput experimental techniques also indicated the shortcomings of high error; not only might the different experimental methods induce different experimental results, but also even different research groups using the same experimental method could not guarantee the exact same result. Therefore, it was urgent to integrate the data from different biological experiments, and even different species, to construct a highly credible network of PPIs. So in this paper, a Bayesian hierarchical model of human PPI network was constructed with a variety of sources of protein interaction data. Meanwhile, a Monte Carlo expectation maximization algorithm was used to estimate the parameters of the model. Then the confidence of protein interaction relationship was calculated based on Bayesian model, and human PPI network with high-confidence level could be obtained.

Thereafter, the role of intrinsic disordered proteins (IDPs) was investigated in the high-confidence PPI network. First of all, different functional modules were obtained through clustering of high-confidence PPI network based on the network topology structure. Then we found the functional modules which were significantly correlated with intrinsically disordered proteins and analysed the effect of IDPs in these functional modules, while searching for the associations between these functional modules and diseases.

## 2. Materials and Methods

### 2.1. Data Collection

In Table 1, we show the experimental data that will be used for the construction of the human PPI network [8–20]. Note that the literature or text mining approach represents most of the low-throughput experimental studies of individual protein-protein interaction. It is possible that the result from the same experiment will be recorded in multiple databases. We will eliminate this type of redundancy. It should be emphasized that the MPC experiments provide result in the format of protein complexes instead of pair-wise protein-protein interactions. Since proteins located in the same complex might not interact with one another directly, we will account for this factor in our model.

### 2.2. Statistical Modeling of Various Data Sources

**Figure 1:**
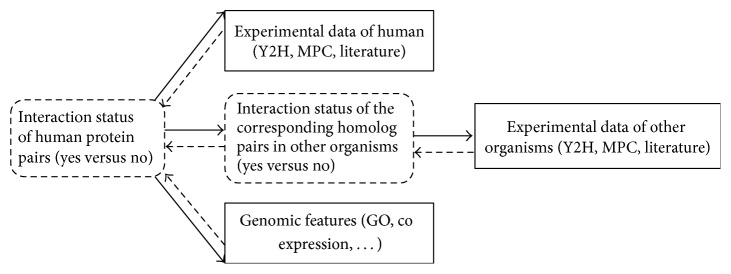
Overall scheme to construct the human protein-protein interaction network. The interaction status of a given pair of human proteins and their homolog in other organisms are unobserved (dashed box) and the experimental data and genomic features are observed evidence (solid boxes). Solid arrows represent model hierarchy and dashed arrows represent inference steps.

The overall scheme of our approach is illustrated in Figure 1. We consider an empirical Bayes approach to integrate various sources of evidence. Let *Z*_*ij*_ be the binary indicator such that *Z*_*ij*_ = 1 means that human proteins *i* and *j* have a direct physical interaction and it is 0 otherwise. Hence, *Z*_*ij*_ is the true interacting status that is not observed. To infer *Z*_*ij*_, we consider individual model for each type of observed data and integrate the evidence to compute the probability of *Z*_*ij*_ = 1.

#### 2.2.1. Human Y2H Data

It has been found that there are a number of mechanisms that can lead to the expression of the reporter gene in a Y2H experiment, which means that an observed interaction might not necessarily mean a true interaction. In our model, we consider the following mechanisms: (a) true interaction; (b) self-activation; and (c) unknown process. Let *Y*_*ij*_ be the binary indicator such that *Y*_*ij*_ = 1 if proteins *i* and *j* are observed to interact in a Y2H experiment and it is 0 otherwise. Then *Y*_*ij*_ = 1 only if at least one of the three above mechanisms is functional. Let *X*_*i*_ = 1 if protein *i* is a self-activation protein and let it be 0 otherwise. We define

(1) αI=Pr⁡a  is  functional ∣ Zij=1,

(2) αS=Pr⁡b  is  functional ∣ Xi+Xj>0,

(3) αU=Pr⁡c  is  functional.

Then we have

(4) Pr⁡Yij=1 ∣ Z,X=1−1−αIZij1−αSXi+Xj1−αU.

#### 2.2.2. Human MPC Data

MPC experiment reveals protein complexes instead of individual pairwise PPI. We say protein B is an *n*-step neighbour of protein A if the shortest path between A and B in the PPI network is of length *n*. We conjecture that the bait will mostly fish out its 1-step neighbours, and 2-step neighbours and distant proteins (at least three step-away) are occasionally observed. Hence, we define the following parameters for the bait proteins:

(5) Pr⁡1-step  neighbour  is  observed=ψ1,Pr⁡2-step  neighbour  is  observed=ψ2.

Let *C*_*k*_ be the set of proteins in a complex corresponding to bait protein* k*. Denote by *n*_*k*_^(1)^, *n*_*k*_^(2)^ the set of 1-step and 2-step neighbours of the bait protein *k* under a given value of *Z*. Then the probability of observing *C*_*k*_ can be written as follows:

(6) Pr⁡Ck ∣ Z=ψ1nk1∩Ck1−ψ1nk1∖Ckψ2nk2∩Ck1−ψ2nk2∖Ck,

where |·| is the function that maps a set to its size.

#### 2.2.3. Literature Data on Human PPI

Let *L*_*ij*_ be the interaction status of proteins *i* and *j* reported. We will account for the false positive rate (*γ*_0,*k*_) and false negative rate (*γ*_0,*k*_):

(7) $$\begin{array}{c} \mathrm{Pr}\left[\;H_{ij}=1\;|\;Z_{ij}\;\right]=\gamma_{1}^{Z_{ij}}\gamma_{0}^{1-Z_{ij}}. \end{array}$$

#### 2.2.4. Data from Other Organisms

We will also collect (*Y* ^*∗*^, *C* ^*∗*^) from other organisms with corresponding unobserved variables denoted by (*Z* ^*∗*^, *X* ^*∗*^). Similar models can be used to model (*Y*, *C*, *L*) for inference of (*Z* ^*∗*^, *X* ^*∗*^). To connect (*Z* ^*∗*^, *X* ^*∗*^) to (*Z*, *X*), we consider the following models:

(8) Pr⁡Zi′j′∗=1 ∣ Zij=Δ1Jii′,jj′;ϕ1ZijΔ0Jii′,jj′;ϕ01−Zij,Pr⁡Xi′∗=1 ∣ Xi=Ω1Iii′;λ1XiΩ0Iii′;λ01−Xi,

where *J*_*ii*′,*jj*′_ is the joint sequence identity between *i* and *i*′ and between *j* and *j*′ and *I*_*ii*′_ is sequence identity between *i* and *i*′; Δ_1_, Δ_0_, *Ω*_1_, and *Ω*_0_ are functions of the joint or individual sequence identities with parameters *ϕ*_1_, *ϕ*_0_, *λ*_1_, and *λ*_0_, which can be modeled by parametric structure.

### 2.3. Construction of Hierarchical Bayesian Model

So far we have introduced the distribution models for the experimental data and genomic features that are conditional on the values of* Z* and* X*. To finish the model, we also need to specify the distributions of* Z* and* X*, which can be modeled with independent Bernoulli distributions:

(9) Pr⁡Zij=1=ρ,Pr⁡Xi=1=r.

With the observed data and the unobserved variables, we can infer the posterior probability of *Z* using the EM algorithm. Note that there are multiple organisms and multiple data sets for some of the organisms. Different parameters will be used to account for difference in the data.

As illustrated in (10), the complete log likelihood function of our model can be expanded below, and the factor of (10) can be substituted by (3)~(9):

(10) LCΨ=fH,Y,W,Z,X,L,Y∗,W∗,Z∗,X∗ ∣ θ=fH ∣ Z,θfY ∣ Z,X,θfW ∣ Z,θfL ∣ Z,θ·fY∗ ∣ Z∗,X∗,θfW∗ ∣ Z∗,θfZ∗,X∗ ∣ Z,X,θfZ,X ∣ θ=∏i,j∈SHfHij ∣ Zij,θ·∏i,j∈SY∏t=1rijfYijt ∣ Zij,Xi,Xj,θ∏i,j∈SM∏t=1eijfWijit ∣ Zij,θ∑t=1ejifWijjt ∣ Zij,θ·∏i,j∈SY∗∏t=1rij∗fYijt∗ ∣ Zij∗,Xi∗,Xj∗,θ∏i,j∈SM∗∏t=1eij∗fWijit∗ ∣ Zij∗,θ∑t=1eji∗fWijjt∗ ∣ Zij,θ∏i,j∈SLfLij ∣ Zij,θ·∏i,j∈S∗fZij∗ ∣ Zij,θ∏i∈SY∗fXi∗ ∣ Xi,θ∏i,j∈SfZij ∣ θ∏i∈SYfXi ∣ θ=∏i,j∈SY1−1−αIZij1−αSXi+Xj1−αUYij+1−αIZij1−αSXi+Xj1−αUYij#·∏i,j∈SHγ1Zijγ01−ZijHij1−γ1Zijγ01−Zij1−Hij∏i,j∈SMψ1ZijWij+1−ψ1ZijWij#×ψ21−ZijWij+1−ψ21−ZijWij#·∏i,j∈SY∗1−1−αIZij∗1−αSXi∗+Xj∗1−αUYij+∗1−αIZij∗1−αSXi∗+Xj∗1−αUYij#∗·∏i,j∈SM∗ψ1Zij∗Wij+∗1−ψ1Zij∗Wij#∗×ψ21−Zij∗Wij+∗1−ψ21−Zij∗Wij#∗∏i,j∈SHγ1Zijγ01−ZijHij1−γ1Zijγ01−Zij1−Hij·∏i,j∈SLβ1Zijβ01−ZijLij1−β1Zijβ01−Zij1−Lij∏i,j∈SρZij1−ρ1−Zij∏i∈SYrXi1−r1−Xi·∏i,j∈S∗ϕ1Zijϕ01−ZijZij∗1−ϕ1Zijϕ01−Zij1−Zij∗∏i∈SY∗λ1Xiλ01−XiXi∗1−λ1Xiλ01−Xi1−Xi∗,

where the parameter vector *θ* = {*ρ*, *r*, *α*_*I*_, *α*_*S*_, *α*_*U*_, *ψ*_1_, *ψ*_2_, *γ*_1_, *γ*_0_, *β*_1_, *β*_0_, *ϕ*_1_, *ϕ*_0_, *λ*_1_, *λ*_0_}.

### 2.4. Monte Carlo Expectation Maximization for Parameter Estimation

In the model, it was not possible to estimate the true value of potential variables and model parameters directly. In order to effectively estimate the potential variables and model parameters, this paper used the Monte Carlo expectation maximization algorithm based on incomplete parameter estimation, as illustrated in Algorithm 1.

In the *E*-step of Algorithm 1, we use Gibbs sampling to sample (*Z*, *X*, *Z* ^*∗*^, *X* ^*∗*^) from $f\left(Z,X,Z^{*},X^{*}|H,Y,W,L,Y^{*},W^{*},{\hat{\theta }}_{0}\right)$ in turn. Repeat the sampling process until the estimations of missing data are obtained. Then in the *M*-step of Algorithm 1, the parameter vector *θ* = {*γ*_1_, *γ*_0_, *α*_*I*_, *α*_*S*_, *α*_*U*_, *β*_1_, *β*_0_, *ϕ*_1_, *ϕ*_0_, *λ*_1_, *λ*_0_} is estimated by Greedy Hill Climbing. Finally the iteration is stopped when diff > 0.01.

## 3. Results

All the protein names were mapped to the Entrez IDs. Finally we got 32540 proteins, and there were 144603 interactions between these proteins.

### 3.1. Construction of the Human PPI Network with Reliable Confidence Measure

Four models were established separately using high-throughput Y2H experimental data, high-throughput MPC experimental data, human PPI data, and all the PPI data. The comparisons among these four models were listed in Table 2.

After the estimation of parameter vector *θ* by Monte Carlo EM, we recalculated the posterior probability of *Z*, which is Pr⁡[*Z*∣*H*, *Y*, *W*, *L*, *Y* ^*∗*^, *W* ^*∗*^], with *θ* and the observed values *H*, *Y*, *W*, *L*, *Y* ^*∗*^, *W* ^*∗*^. And for each pair of PPI, we considered them as reliable confidence interaction if Pr⁡[*Z*_*ij*_ = 1∣*H*, *Y*, *W*, *L*, *Y* ^*∗*^, *W* ^*∗*^] > 0.8. Then we got 48361 PPIs with reliable confidence measure among 23286 proteins.

### 3.2. Characterization of Network and Roles of IDPs Based on Network Topology

We analysed the role of IDPs in the human PPI networks with reliable confidence measure. A IDP was defined as a protein with continuous intrinsically disorder region whose length was larger than 40 amino acids. And 8735 IDPs were identified from 23286 proteins after predictions.

Firstly, the human PPI network was cut into subnetworks or modules by SCAN. SCAN obtained modules based on the similarity between common neighbors. Then we used modularity and similarity-based modularity as metrics. Modularity is a statistical measure of the quality of network clustering, which is defined as follows:

(11) $$\begin{array}{c} Q_{N}=\overset{N_{C}}{\underset{s=1}{\sum }}\left[\;\frac{l_{s}}{L}-{\left(\;\frac{d_{s}}{2L}\;\right)}^{2}\;\right], \end{array}$$

where *N*_*C*_ is the number of clusterings,* L* is the number of edges, *l*_*s*_ is the number of edges for *s*_th_ module, and *d*_*s*_ is the degree of all the nodes in *s*_th_ module. We could obtain the best clustering by optimizing *Q*_*N*_. And similarity-based modularity is the supplementary for the modularity, which is defined as follows:

(12) $$\begin{array}{c} Q_{S}=\overset{NC}{\underset{s=1}{\sum }}\left[\;\frac{IS_{i}}{TS}-{\left(\;\frac{DS_{i}}{2TS}\;\right)}^{2}\;\right]. \end{array}$$

**Figure 2:**
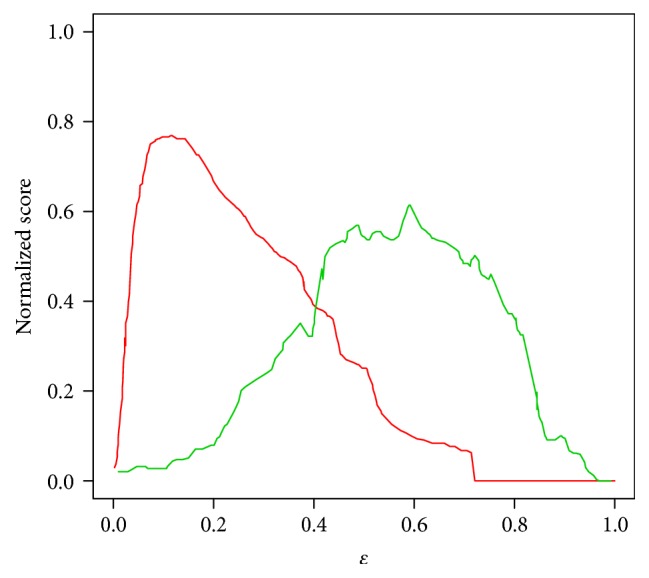
The optimization of *Q*_*N*_ and *Q*_*S*_ for different *ε*. Red line and green line correspond to *Q*_*N*_ and *Q*_*S*_ separately.

As shown in Figure 2, on one hand, the modularity monotonically decreased from the position nearby zero, and it could not be maximized. On the other hand, the similarity-based modularity could be maximized while the threshold *ε* equals 0.61. Conditional on the *ε* = 0.61, the reliable human PPI network was cut into 241 modules. Under the significant level *α* = 0.05, the *p* value of each module was calculated by the formula below:

(13) $$\begin{array}{c} p\text{-value}=\overset{n}{\underset{i=m}{\sum }}\frac{\left(\begin{array}{c} M\\ i \end{array}\right)\left(\begin{array}{c} N-M\\ n-i \end{array}\right)}{\left(\begin{array}{c} N\\ n \end{array}\right)}, \end{array}$$

where *N* is the number of all the proteins and *M* is the number of all the IDPs. 33 modules among 241 modules were significantly associated with IDPs.

**Figure 3:**
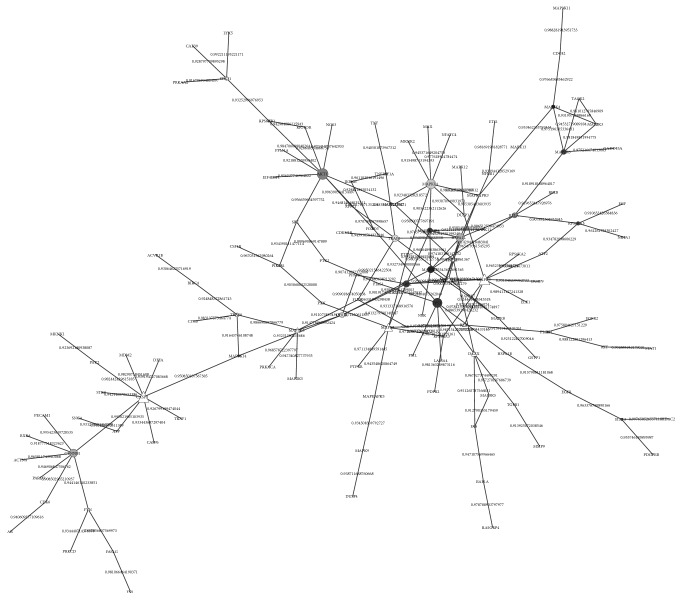
A reliable subnetwork for hela cell. Circles correspond to IDPs. And the degree of grey corresponds to the length of intrinsically disordered region for IDP.

However, due to the fact that acquisition of functional modules is only dependent on the network topology, we analysed the modules with known diseases. And the overlap of PPI in hela cell and a functional module which was highly related with IDPs was shown in Figure 3. The weight of each side is the posterior probability of the real value* Z*. If a node with more than 5 neighbours was defined as a hub node in this subnetwork, a total of 69% of the hub nodes were IDPs. It is verified that IDPs were easy to become hub nodes of the protein interaction network due to the flexibility of the structure, revealing an important role of IDPs in the regulation of cervical cancer hela cell.

## 4. Discussion

Our model is unique and novel in the following perspectives. First, it integrates Y2H and MPC data in a cohesive and unified model that connect the two types of data through the unobserved true status of direct physical interaction *Z*. Second, the model allows a natural calculation of the confidence of each interacting pair via the posterior probability. This is a critical measurement in downstream analysis and will be accounted for. To our knowledge, no previous study has considered uncertainty in the PPI network analysis.

The inference of the interacting probability involves a large number of latent variables. The combinatorial effects make it impractical to compute the expectation of the missing variables analytically during the *E*-step. It is likely that various data sets carry different amount of information regarding the true interaction status. Hence, the inference can be made by appropriately weighing data of various types instead of treating them equally. This can be achieved by setting parameter constrain.

## Figures and Tables

**Algorithm 1:**
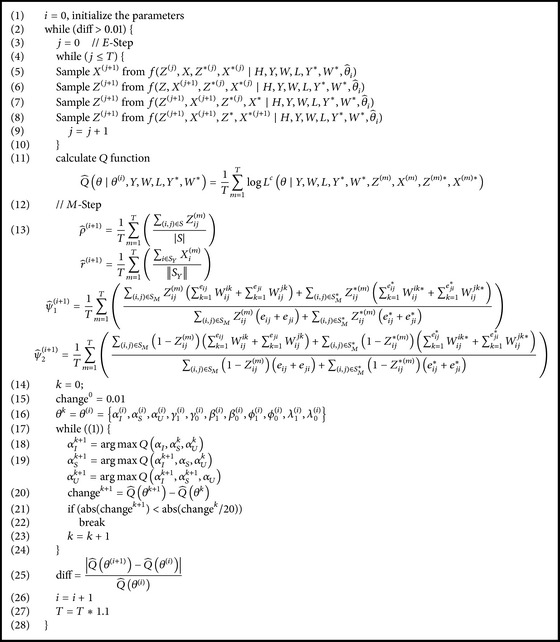
Monte Carlo expectation maximization for parameter estimation.

**Table 1:** Data sets or databases used to construct the human protein-protein interaction network.

Method	Organism	Reference
Y2H	Human	Stelzl et al. [8]
Y2H	Human	Rual et al. [9]
MPC	Human	Ewing et al. [10]
Literature	Human	HPRD [11], http://www.hprd.org/
Y2H	Yeast	Ito et al. [12]
Y2H	Yeast	Uetz et al. [13]
MPC	Yeast	Gavin et al. [14]
MPC	Yeast	Ho et al. [15]
MPC	Yeast	Gavin et al. [16]
MPC	Yeast	Krogan et al. [17]
Literature	Multiple	IntAct [18], http://www.ebi.ac.uk/intact/
Literature	Multiple	MIPS [19], http://mips.gsf.de/proj/ppi/
Multiple	Multiple	DIP [20], http://dip.doe-mbi.ucla.edu

**Table 2:** Comparison of parameters based on different data.

Parameters	High-throughput Y2H	High-throughput MPC	Human PPI data	All PPI data
*ρ*	6.8 × 10^−3^	1.9 × 10^−3^	6.1 × 10^−3^	1.4 × 10^−2^
*r*	7.7 × 10^−5^	—	5.3 × 10^−5^	8.9 × 10^−5^
*α*_*I*_	0.658	—	0.543	0.933
*α*_*S*_	0.426	—	0.496	0.852
*α*_*U*_	4.5 × 10^−3^	—	9.7 × 10^−4^	0.007
*ψ*_1_	—	0.738	0.755	0.809
*ψ*_2_	—	0.623	0.764	0.788
